# Overview on HTLV-1 p12, p8, p30, p13: accomplices in persistent infection and viral pathogenesis

**DOI:** 10.3389/fmicb.2012.00400

**Published:** 2012-12-11

**Authors:** Xue Tao Bai, Christophe Nicot

**Affiliations:** Department of Pathology and Laboratory Medicine, University of Kansas Medical CenterKansas City, KS, USA

**Keywords:** HTLV-1, pathogenesis, immune response, oncogenesis, virus replication

## Abstract

The human T-lymphotropic virus type-1 (HTLV-1) is etiologically linked to adult T cell leukemia/lymphoma and tropical spastic paraparesis/HTLV-1-associated myelopathy. While the role of Tax and Rex in viral replication and pathogenesis has been extensively studied, recent evidence suggests that additional viral proteins are essential for the virus life cycle *in vivo*. In this review, we will summarize possible molecular mechanisms evoked in the literature to explain how p12, p8, p30, and p13 facilitate persistent viral infection of the host. We will explore several stratagems used by HTLV-1 accessory genes to escape immune surveillance, to establish latency, and to deregulate cell cycle and apoptosis to participate in virus-mediated cellular transformation.

## INTRODUCTION

Expression of viral proteins and replication is under the control of virus long terminal repeat (LTR) transactivator Tax and viral RNA export Rex proteins (Kashanchi and Brady, 2005). The regulation of human T-lymphotropic virus type-1 (HTLV-1) structural protein(s) expression and their processing has been previously reviewed (Le Blanc et al., 2001; Fogarty et al., 2011). Unlike animal oncoretroviruses the HTLV-1 proviral genome encodes a unique pX region, which, through alternative splicing and different translation initiation sites, generates seven viral regulatory proteins (Ciminale et al., 1992; Koralnik et al., 1993). This complexity is remarkable and highlights the high level of adaptation the virus employs in making the most out of its relatively small genome. All regulatory proteins are expressed in HTLV-1 cell lines and in patients infected with HTLV-1 (Pique et al., 2000; Satou et al., 2006; Saito et al., 2009). Expression of these proteins is absolutely essential for virus replication in an *in vivo* non-human primate model for HTLV-1 (Valeri et al., 2010).

Open reading frame-I (ORF-I) encodes the p12 protein, a small hydrophobic protein which can be further processed into p8 (Koralnik et al., 1993). p12 and p8 have different cellular localizations and distinct functions (Van Prooyen et al., 2010a). Recent studies indicate that some of the functions once attributed to p12 actually belong to p8. ORF-II produces the p30 and p13 proteins. Although p13 encodes the carboxyl terminal 87 amino acids of p30, their cellular localizations and functions are significantly different (Koralnik et al., 1993; Baydoun et al., 2008; Silic-Benussi et al., 2010a). Rex and Tax, corresponding to ORF-III and ORF-IV, are encoded by the same doubly spliced RNA (Kashanchi and Brady, 2005). HBZ is encoded from a complementary minus-stranded RNA transcript from the 3′-LTR (Gaudray et al., 2002; Murata et al., 2006). In addition, the pX region also produces two other proteins, p21-Rex and Rof (p12 Rex Orf I). This review will focus on the role of HTLV-1 regulatory genes in virus infection, immune escape, and transformation.

## ROLE OF HTLV-1 REGULATORY GENES IN VIRUS INFECTION AND VIRAL SPREADING *IN VIVO*

In contrast to Tax and Rex, HTLV-1 regulatory genes p12, p8, p30, and p13 are not absolutely required for virus replication and for the immortalization of human primary T cells *in vitro* (Derse et al., 1997; Robek et al., 1998; Lairmore et al., 2000). However, several studies have found that human T cell lines immortalized with HTLV-1 molecular clones lacking p12 or p30 grow less efficiently than their wild-type counterpart clones and are more dependent upon the presence of interleukin-2 (IL-2) in the media (Albrecht et al., 2000; Nicot et al., 2001; Taylor et al., 2009a). Investigations using a rabbit model to study HTLV-1 infection *in vivo* suggested that p12, p30, and p13 may all be required for viral infectivity (Bartoe et al., 2000; Silverman et al., 2004; Hiraragi et al., 2006). However, the subsequent discovery of a new viral gene, HBZ, encoded from an overlapping complementary minus-stranded RNA transcript from the 3′-LTR, challenged conclusions made in these studies. Mutations aimed at knocking out p12, p30, and p13 in HTLV-1 molecular clones all affected the HBZ coding sequence, a gene necessary for virus infectivity (Matsuoka and Jeang, 2007; Yasunaga and Matsuoka, 2011). Recently, the role of HTLV-1 regulatory genes was re-investigated using new HTLV-1 molecular clones carrying a single viral gene knock-out, whereby HBZ expression was not affected. These studies, which were performed both in rabbits and in non-human primates, clearly demonstrated that only non-human primates constitute an appropriate *in vivo* model for HTLV-1 infection and replication (Valeri et al., 2010).

HBZ, p12, and p30 were found to be essential for HTLV-1 infection and replication in non-human primates but p12 and p30 were dispensable in rabbits (Valeri et al., 2010). Interestingly, it appears that the requirement of p12 and p30 for infectivity *in vivo* is related to their ability to sustain HTLV-1 replication in dendritic cells (DCs) in *in vitro* and *in vivo* infection of macaques (Valeri et al., 2010). These results also confirmed earlier studies showing that HTLV-1 infection of DCs is essential for initial viral spread (Jones et al., 2008). These observations are very critical in understanding the development of adult T cell leukemia/lymphoma (ATLL), because HTLV-1 must spread rapidly to its target cells to establish a latent infection and avoid clearance by the immune system.

Human T-lymphotropic virus type-1 cell free virions are poorly infectious and require cell-to-cell transmission. In this regard it is interesting to point out studies that have demonstrated a role for p12 in facilitating cell-to-cell viral spread by inducing lymphocyte function-associated antigen-1 (LFA-1) clustering on T cells. These effects relied upon calcium-dependent signaling and increased nuclear factor of activated T cells (NFAT)-dependent transcription (Kim et al., 2006). Therefore, p12 may play an essential role in the very early stages of HTLV-1 infection.

## TRANSCRIPTIONAL REGULATION OF VIRAL EXPRESSION BY REGULATORY GENES

**FIGURE 1:**
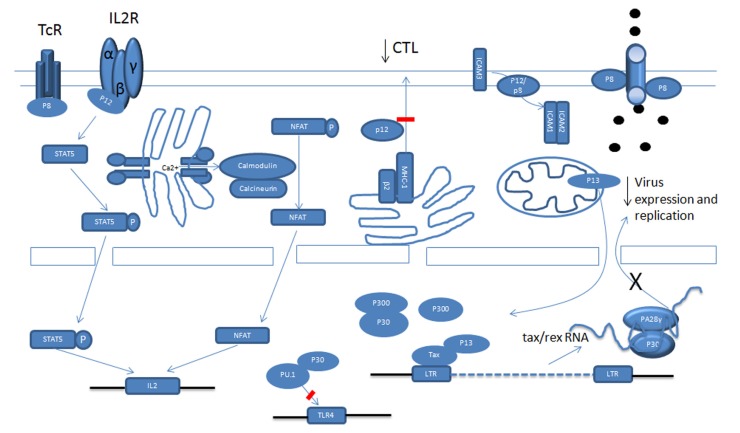
**Schematic functional representation of p12, p8, p30 and p13 in HTLV-1 infection**.

Once the HTLV-1 virus establishes infection it is critical for the virus to repress viral gene expression in order to reduce viral antigens and avoid being recognized by the immune system. Tax and Rex are potent positive regulators of viral expression and Tax is also the main target of cytotoxic T lymphocytes (CTLs; Koenig et al., 1993). Because of this, the HTLV-1 virus employs different ways to interfere with Tax function and to reduce Tax expression. p30, p13, and p8 all negatively regulate viral expression (**Figure 1**; Nicot et al., 2004; Andresen et al., 2011). Tax activates the transcription of viral genes through the recruitment of cAMP response element-binding protein (CREB) and cellular coactivators CREB-binding protein (CBP), p300 and p300/CBP-associated factor (PCAF) to the Tax-response elements (TRE) within the U3 region of the LTR (Kashanchi and Brady, 2005). Some studies have demonstrated that p30 has the ability to attenuate the formation of active transcriptional complexes on the TRE and can interact with CBP/p300 via the KIX domain (Zhang et al., 2001). Because Tax recruits CBP/p300 via the same domain, Tax and p30 are proposed to compete with each other for CBP/p300 binding. However, these observations were made in an over-expression experimental system and are therefore subject to discrepancy (Nicot et al., 2004). In another study, whereby p30 was expressed from an HTLV-1 molecular clone, p30 could not decrease virus production (Valeri et al., 2010). This is expected when the expression level of Tax and p30 are taken into account, as there is much more Tax than p30 mRNA and proteins in HTLV-1 transformed cells *in vitro* than in transiently transfected cells, which may not accurately reflect the expression levels of these proteins *in vivo*. The relative level of these proteins in ATLL cells in still unknown and may also vary in distinct cellular sub-populations. A recent study showed the expression level of *p30* was about 1/1000 of *tax/rex* RNA in ATLL samples after *in vitro* culturing for 2 h (Rende et al., 2011). However, this ratio might not reflect the actual ratio *in vivo.*

In addition to viral gene transcription, genome-wide analysis revealed that p30 modulates the transcription of numerous cellular genes. Sixty-five genes were found to be down-regulated greater than 2.5-fold in the presence of p30. These genes were found to be associated with cell signaling, transcription/translation, cell cycle, and metabolism (Taylor et al., 2009b). In contrast to repressed genes, only 15 cellular genes were up-regulated and mostly corresponded to genes involved in transcription/translation and RNA processing (Taylor et al., 2009b).

A recent study showed that p13 is also able to interfere with Tax transactivation. Using GST pull-down and co-immunoprecipitation assays, p13 was shown to interact with Tax, and its presence can decrease Tax’s interaction with p300. Interestingly, the fact that an increasing amount of p300 can partially rescue the repression by p13 on the HTLV-1 LTR reporter also indicates a competition mechanism. In fact, p13 and CBP/p300 have been shown to compete for Tax interaction. Notably, even when p13 was expressed naturally from an HTLV-1 molecular clone, it can still repress viral replication. The difference between p13 and p30 mediation of Tax transactivation might be caused by two factors. Firstly, a previous study showed that in transient transfection with an HTLV-1 molecular clone, there was more p13 expression than p30 expression. Secondly, the stability of p13 can be greatly increased by Tax. In addition to p30 and p13, studies show that a p12-processed p8 protein can also decrease viral transcription through mediation of T cell receptor (TCR) signaling. Previous studies demonstrate that activation of the TCR increases viral replication. The TCR complex consists of a variable ligand-binding TCRαβ heterodimer and a non-variable signal transduction CD3 complex including CD3γ, CD3δ, CD3ε, and a TCRζ subunit. TCR signaling occurs through a cascade of events. It is not clear how the TCR pathway activates viral transcription, but some evidence suggests that lymphocyte-specific protein tyrosine kinase (Lck) plays a significant role in transduction. Studies have demonstrated that p8 inhibits TCR signal transduction and reduces viral expression. When the non-canonical endoplasmic reticulum (ER) retention/retrieval signal (1–9 aa) within the amino terminus is removed from p12, the protein was transported to the Golgi apparatus, where another cleavage between amino acids 29 and 30 produced p8 and stimulated its expression at the cell surface. When the TCR is activated and forms the immunologic synapse (IS), p8 is quickly recruited to the IS. In the IS, p8 binds to the linker of activated T cells (LAT) protein and inhibits its activity, as evidenced by the decreased phosphorylation of phospholipase C-gamma1 (PLC-γ1) and Vav. A similar inhibition was observed when p8 was expressed from an HTLV-1 molecular clone.

## POST-TRANSCRIPTIONAL REGULATION OF VIRAL EXPRESSION BY REGULATORY GENES

In addition to transcription, prior studies show that p30 can negatively regulate viral expression after transcription. We found that p30 binds to a response element within the *tax/rex* RNA and retains the viral RNA in the nucleus. This further reduces Tax expression and silences virus expression (Nicot et al., 2004, 2005). When over-expressed, p30 efficiently represses viral replication in both transient transfection assays and HTLV-1-infected cells, such as MT2 and C91PL (Nicot et al., 2004). Interestingly, recent studies demonstrated that ablation of p30 from an HTLV-1 molecular clone reduced replication; and HBZ suppressed HTLV-1 replication by targeting p30 mRNA (Choudhary and Ratner, 2011).

We recently found that p30 interacts with PA28gamma (PA28γ) and recruits it to the *tax/rex* RNA. The binding of PA28γ to *tax/rex* RNA is p30-dependent. In 293FT cells knocked-down for PA28γ expression, viral production from transfected HTLV-1 molecular clones significantly increased. In an HTLV-1 positive ATLL cell line, the knock-down of PA28γ increased *tax/rex* RNA and Tax proteins. These studies support the notion that endogenous p30 expressed under physiological conditions from the provirus can regulate *tax/rex* expression.

## ROLE OF HTLV-1 SMALL REGULATORY GENES IN HOST IMMUNE ESCAPE AND IMMUNOSUPPRESSION

There is abundant evidence that HTLV-1-specific CTLs are efficient against HTLV-1-infected cells and play a significant role in determining the proviral load (Bangham, 2000; Bangham and Osame, 2005; Akimoto et al., 2007; Bangham et al., 2009). So far, there is evidence that most of the viral proteins can be targeted by CTLs. However, it is generally accepted that the dominant target of CTLs is the Tax protein. In addition, natural killer (NK) cells can also kill HTLV-1-infected cells. Despite this, HTLV-1 is still able to establish persistent infection in its host. Studies show that the virus employs different strategies to remain silent. First, it represses viral gene expression, while persisting in the host through proliferation of infected cells (Saggioro et al., 1991; Newbound et al., 2000; Rahman et al., 2012). Second, the virus has also evolved ways to interfere with the immune response of the host.

### p8 STIMULATES FORMATION OF VIRAL TUNNELS FOR INFECTION OF NEIGHBORING CELLS

Although there is evidence that cell-free HTLV-1 virus can infect DCs, it is generally accepted that HTLV-1 is mainly transmitted through cell-to-cell contacts, such as virological synapses and cellular conduits (Nejmeddine et al., 2005). Studies show that p8 increases virus transmission between cells through cellular conduits. Studies showed that p8 enhances cell adhesion by inducing LFA-1 clustering without changing LFA-1’s expression or affinity (Van Prooyen et al., 2010b). Immunofluorescence demonstrated that p8 co-localized with the clustered LFA-1. It was further proved that p8 expressed from the HTLV-1 molecular clone had the same effects (Van Prooyen et al., 2010b). The HTLV-1 virus traffics through the conduits, as evidenced by the presence of mature viral particles at the contact site between two conduits or between a conduit and the surface of the target T cell. Collectively, p8 provides the virus with a new way to infect cells hiding from immune defenses (Van Prooyen et al., 2010b).

### DOWN-MODULATION OF MHC-1 BY p12

Cytotoxic T lymphocytes target HTLV-1-infected cells through the TCR, which recognizes the viral peptide presented by the major histocompatibility complex class I (MHC-1). The MHC-1 consists of a heavy chain (Hc) containing the peptide binding site and β2-microglobulin, which are assembled in the lumen of the ER. Viral peptides, generated by the proteasome, are transported into the ER, where the three components assemble in ternary complexes and are transported to the cell surface. The abnormality of the assembly and trafficking of MHC-1 helps the infected cells to evade recognition by the CTL, contributing to viral persistence. Studies show that p12 interferes with the assembly and decreases from the cell surface MHC-1. p12 specifically binds to newly synthesized, less glycosylated MHC-1-Hc before it forms a heterodimer with β2-microglobulin (Johnson et al., 2001; Johnson and Franchini, 2002). Three different human MHC-1-Hc-A2, B7, and Cw4 complexes were tested and all of them interacted with p12. Upon binding, the MHC-1-Hc and p12 complex is rerouted to the cytosol and degraded by the proteasome. Cellular immunofluorescence demonstrated that p12 also interfered with the trafficking of MHC-1 and, consequently, the level of MHC-1 on the cell surface decreased. In addition, in the presence of p12, a decrease of endogenous MHC-1 was also demonstrated (Johnson et al., 2001). Moreover, the MHC-1 molecules were decreased after primary CD4^+^ T cells were infected by the HTLV-1 virus (Johnson et al., 2001).

### REDUCTION OF ICAM-1 AND ICAM-2 BY p12

As stated above, the HTLV-1 virus down-regulates cell surface MHC-1 to evade CTLs. At the same time, the down-regulation of MHC-1 may expose the infected cells to NK cells. It is also known that Tax increases IL-2 expression (Ballard et al., 1988; Ruben et al., 1988), which promotes NK cell proliferation and enhances LFA-1-mediated adhesion of NK cells to intercellular adhesion molecules (ICAMs) on target cells. Taken together, this suggests that NK cells can easily adhere to and kill HTLV-1-infected cells (Stewart et al., 1996). Surprisingly, studies show there was little difference between NK cell cytotoxicity of the mock-infected cells and the HTLV-1-infected cells. Also, pretreatment of cells with IL-2 just marginally increased NK cell cytotoxicity to HTLV-1-infected cells. Further studies demonstrated that HTLV-1 infection specifically decreased the expression of ICAM-1 and ICAM-2, but not ICAM-3 on primary T cells (Banerjee et al., 2007). Moreover, it was demonstrated that expression of p12 alone is sufficient to down-modulate them (Banerjee et al., 2007). In addition, lack of natural cytotoxicity receptor (NCR) and NKG2D ligand expression from HTLV-1-infected cells might also affect NK cell activity. So far, it is not clear whether p12 or p8 mediate the down-modulation of ICAM-1 and ICAM-2, and the mechanism for doing so also remains to be seen.

### INHIBITION OF TLR-4 SIGNALING BY p30 IN MACROPHAGES

Although the primary target of HTLV-1 is CD4^+^ cells, many other kinds of cells can also be infected both *in vivo* and *in vitro*, such as monocytes, macrophages, and DCs (Jain et al., 2009). These cells are important for innate immunity and play a major role in antigen presentation. There are some reports stating that infection by HTLV-1 interferes with the differentiation and function of DCs. Our studies show that p30 interferes with Toll-like receptor-4 (TLR-4) signaling in human macrophages (Datta et al., 2006; Bai et al., 2010). TLR-4 is the major lipopolysaccharide (LPS) receptor and elicits an innate immune response against Gram-negative bacteria. We found that p30 binds to and inhibits PU.1 DNA binding and that p30 inhibits endogenous PU.1-mediated transcription from a PU.1 reporter in human macrophages (Datta et al., 2006). In addition, p30 reduces endogenous PU.1 expression. The inhibition of PU.1 by p30 was further evidenced by a decrease in TLR4 expression (Datta et al., 2006). Notably, when p30 was expressed from an HTLV-1 molecular clone, it still down-regulated TLR-4 expression. Consistent with this, in the presence of p30, the release of pro-inflammatory cytokines monocyte chemotactic protein-1(MCP-1), tumor necrosis factor-alpha (TNF-α), and IL-8 decreased after stimulation with LPS (Datta et al., 2006). Although innate and adaptive immune responses have been thought to be non-overlapping, recent evidence clearly indicates that the interplay between components of the immune system occurs frequently and forms the basis of effective immunity. The impact of inhibition of PU.1 by p30 on the host immune response remains to be studied.

Recent studies highlight another side of macrophage and DC infection. They are not just “victims” of the HTLV-1 virus, but may actually help virus transmission, dissemination and persistence (Jones et al., 2008). The importance of these observations remains to be clarified. Interestingly, an HTLV-1 molecular clone with p12 or p30 ablation cannot infect human primary DCs, suggesting that they play an essential role in DC infection (Valeri et al., 2010).

## HOW HTLV-1 REGULATORY GENES STIMULATE T CELL PROLIFERATION, AFFECT DNA REPAIR, AND PROMOTE CELLULAR TRANSFORMATION

### ACTIVATION OF STAT5b BY p12

Interleukin-2 is an important cytokine that drives T cell proliferation. There are three different IL-2 receptors (IL-2R): α chain (IL-2Rα), β chain (IL-2Rβ), and γ chain [IL-2Rγ, also known as the common cytokine receptor γ chain (γ_*c*_)]. Ligand-specific IL-Rα (CD25) is expressed on activated lymphocytes and binds IL-2 with low affinity. The IL-2Rβ/IL-2Rγ complex binds IL-2 with intermediate affinity. When all three receptors are expressed on activated T cells, IL-2 is bound with high affinity. The intermediate and high affinity receptor forms are responsible for IL-2 signal transduction (Waldmann et al., 1998; Imada and Leonard, 2000). Binding of IL-2 leads to the heterodimerization of the cytoplasmic domain of IL-2Rβ and γ_*c*_, followed by the recruitment of Janus kinase 1 (Jak1) and Jak3. Then, Jak1 and Jak3 activate signal transducers and activators of transcription (STAT) proteins through phosphorylation. IL-2Rβ is essential for STAT protein docking and activation (Waldmann et al., 1998; Imada and Leonard, 2000). In HTLV-1 cells, there exists constitutive activation of the Jak/STAT pathway (Migone et al., 1995, 1998; Xu et al., 1995; Takemoto et al., 1997). Although Tax can activate the Jak/STAT pathway through inducing IL-2, IL-2Rα, and STAT5 expression, the fact that there is little or no Tax expression in the majority of ATLL patient samples indicates that another mechanism exists. p12 has the ability to activate the Jak/STAT pathway. p12 significantly increased the transcriptional activity and DNA binding of STAT5b (Nicot et al., 2001). The activation required the presence IL-2Rβ, γ_*c*_, and Jak3. Forced expression of p12 in peripheral blood mononuclear cells (PBMCs) also resulted in more STAT5 phosphorylation and DNA binding. Consistent with the activation of the Jak/STAT pathway by p12, t p12 decreases the IL-2 requirement for T cell proliferation and promotes cell proliferation by limiting the concentration of IL-2 (Nicot et al., 2001). In addition, p12 also increases the colony formation potential of HTLV-1 with and without IL-2. It is notable that when p12 was expressed naturally in the HTLV-1 molecular clone, it still decreased the cells’ dependency on IL-2 (Nicot et al., 2001).

### p12 DEREGULATES Ca^2+^ AND NFAT

Except for the Jak/STAT pathway, p12 also has the ability to activate another important T cell transcription factor, the NFAT. There are five members in the NFAT family. Among them, NFAT1 to NFAT4 are regulated by intracellular Ca^2+^ signaling and, in activated T cells, NFAT2 is the predominantly induced factor. Purified NFAT is heavily phosphorylated and is dephosphorylated by the phosphatase calcineurin. All kinds of stimuli that activate NFAT will first result in the release of Ca^2+^ from the ER. The released Ca^2+^ in the cytoplasm activates the Ca^2+^ sensor calmodulin, followed by the activation of the calmodulin-dependent phosphatase, calcineurin (Medyouf and Ghysdael, 2008; Oh-hora and Rao, 2009). Calcineurin dephosphorylates multiple phosphoserines from the regulatory domain of NFAT and activates NFAT. The activated NFAT translocates into the nucleus and cooperates with transcriptional factors, including activator protein-1 (AP-1), to activate transcription. p12’s ability to activate NFAT is related to its ability to increase cytoplasmic Ca^2+^ (Albrecht et al., 2002; Ding et al., 2002; Kim et al., 2003). In support of this conclusion, activation can be blocked by BAPTA-AM [glycine, *N,N*′-1,2-ethanediylbis(oxy-2,1-phenylene)-bis-*N*-2-(acetyloxy) methoxy-2-oxoethyl]-[bis(acetyloxy)methyl ester], a sequester of intracellular calcium. How p12 increases cellular calcium is not clear. However, that 2-APB, an inositol 1,4,5-trisphosphate (IP3) receptor, and SKF 96365, a chemical inhibitor of release-activated calcium (CRAC) channels in the plasma membrane, can partially block the activation implies that both ER calcium releasing and extracellular-calcium influx contribute to the cytoplasmic calcium increase. In addition, cyclosporin A and a dominant negative NFAT2 mutant also inhibit the activation by p12. It is also reported that p12 has a calcineurin binding motif PSLP(I/L)T, which is highly homologous to the PXIXIT calcineurin binding motif of NFAT. p12 binds to calcineurin and competes with NFAT for calcineurin binding (Albrecht et al., 2002; Ding et al., 2002; Kim et al., 2003). Because both activated STAT5 and NFAT can bind to the IL-2 promoter and increase transcription, the presence of p12 should enhance IL-2 production. As expected, p12 expression in Jurkat T cells and PBMCs enhances IL-2 production. The enhanced IL-2 production by p12 was calcium-dependent, which suggests that the NFAT pathway might be even more important to p12 in enhancing IL-2 production. In addition, the quantities of IL-2 elicited from p12-expressing PBMCs were sufficient to promote PBMC proliferation. Interestingly, we found that IL-2 increased HTLV-1 virus transmission. We further demonstrated that inhibition of Jak/STAT signaling decreased virus transmission. Transmission was associated with promotion of cell membrane fusion, but not with virus production, cell–cell adherence, gags polarization, or virological synapse formation. This mechanism remains to be clarified. Except for all the advantages of IL-2, it also stimulates CTL proliferation and evokes NK cell cytotoxicity toward HTLV-1-infected cells, helping to eradicate them.

### p30 ALTERS CELL CYCLE AND DNA REPAIR TO PROMOTE TRANSFORMATION

Using microarray analysis, the effect of p30 on gene expression was studied by two different groups (Datta et al., 2007; Taylor et al., 2009b). Based on the changes in gene expression, p30 modulates many aspects of cell function, such as apoptosis, cell cycle, T cell activation and signal transduction, and transcription/translation/RNA processing factors. Overall, p30 down-regulates more genes than it up-regulates. We also studied the effect of p30 on RNA export and found that p30 increased or decreased a large portion of genes in the cytoplasm. The biological significance of these changes remains to be tested. It is of note that all these assays were based on over-expression systems. Based on the array data, p30 was tested on NF-κβ, AP-1, and NFAT reporters with and without co-stimulators of T cells, including phorbol 12-myristate 13-acetate (PMA), ionomycin, anti-CD3, and anti-CD28. p30 activated all three reporters in Jurkat T cells (Datta et al., 2007). Collectively, these data suggested that p30, like p12, has the potential to promote T cell proliferation. However, the role of p30 in regulating the cell cycle of T cells is not yet determined, and so far three studies have had different results. In one study, it was demonstrated that p30 results in a delay in the G2 phase of the cell cycle (Datta et al., 2007). In addition, the p30 transduced Jurkat T cells proliferated slowly compared to the mock-infected cells. The delay was associated with the G2-M transition checkpoint, including phosphorylation of checkpoint kinase-1 (Chk1) at serine 345, phosphorylation of Cdc25C at serine 216, reduced amounts of Polo-like kinase-1 (PLK1) and the threonine 210 phosphorylated form of PLK1 and dephosphorylation of Cdc2 at threonine 198 and 216 (Datta et al., 2007). However, in another study it was found that p30 inhibits G1-S progression and retains cells in G1 in both HeLa and Jurkat T cells (Baydoun et al., 2010). p30 prevents S phase entry by targeting multiple checkpoints, as evidenced by reduced phosphorylation of retinoblastoma protein (Rb), decreased expression of E2F, proliferation cell nuclear antigen (PCNA), and Cyclin E, and increased expression of p21 waf (Baydoun et al., 2010). In addition, we demonstrated that p30 interacts with both CDK2 and Cyclin E and reduces active CDK2–Cyclin E complex formation (Baydoun et al., 2010). Because Tax promotes cell cycle progression, especially at the G1-S phase transition (Franchini et al., 2003), p30 has a new way to negatively regulate Tax. Further studies are needed to understand the function of p30 on cell cycle and the relationship(s) between deregulation of the cell cycle, viral persistence, and leukemogenesis.

In addition to deregulation of the cell cycle, p30 also interferes with DNA repair to promote cancer. In a recent study, it was found that p30 inhibits homologous recombination (HR) repair (Baydoun et al., 2011). When there was a DNA double strand break (DSB) caused by drugs or irradiation, p30 re-localized from the nucleoli to the nucleoplasm. p30 re-localization was associated with phosphorylation of p30 at threonine 232 by the mitogen-activated protein kinase (MAPK) signaling pathway, since re-location was blocked by a threonine to alanine mutation at 232 of p30 and the MAPK inhibitor, PD98059 (Baydoun et al., 2011). Several pieces of evidence indicated that amino acids 221–254 of p30’s c-terminus are not involved in HR. Notably, p30 was demonstrated to interact with both nibrin (NBS1) and RAD50, but not MRE11, and p30 was able to reduce the assembly of functional Mre11–Rad50–NBS1 (MRN) complexes onto DSBs (Baydoun et al., 2011). Moreover, p30 also disrupted the MRN complex formation on naturally occurring DSBs during S-phase. Corresponding to a decrease in HR repair was an increase in error-prone non-homologous end-joining (NHEJ) repair. Collectively, these data suggest that p30 increases the instability of the genome and plays an important role in T cell transformation and leukemogenesis (Baydoun et al., 2011). Our finding is consistent with the facts that p30 protects cells from camptothecin, a topoisomerase I inhibitor, which induces apoptosis in cells in the S phase of the cell cycle, and irradiation. A recent study also showed that p30 binds to ataxia telangiectasia mutated (ATM) and modulates its phosphorylation to prevent apoptosis (Anupam et al., 2011). In addition to increasing genomic instability, p30 also promotes transformation through enhancing the transcriptional and transforming activity of Myc. p30 interacts with both Myc and TIP60 and helps Myc to recruit more TIP60 to the Myc transcriptional complex (Awasthi et al., 2005).

### THE ROLE OF p13 IN PRO-APOPTOSIS AND INHIBITION OF TUMOR CELL PROLIFERATION

p13 localizes to both nuclear speckles and mitochondria depending on the cellular context and expression level (D’Agostino et al., 2000). The amino acids 21–30 of p13, the minimal mitochondrial targeting sequence, are responsible for mitochondria targeting. Studies show that p13 might form an amphipathic α- helix across the inner membrane and trigger an inward K^+^ and Ca^+^ current that causes depolarization, activation of the electron transport chain and augmentation of reactive oxygen species (ROS) production (Silic-Benussi et al., 2009, 2010c; Biasiotto et al., 2010). Further studies showed that even at lower expression levels or through expression in the context of the viral genome, p13 can increase ROS production and induce apoptosis (D’Agostino et al., 2005; Hiraragi et al., 2005). There is evidence that p13 can significantly reduce the incidence and growth rate of tumors arising from c-myc and ha-ras-co-transfected rat embryo fibroblasts (Silic-Benussi et al., 2004). It is not clear if p13 has the same function when it is expressed at lower levels or in its natural context. Interestingly, later studies found that p13 also increased ROS production in normal primary T cells and activated T cells, which was not associated with apoptosis (Silic-Benussi et al., 2010b). The effect was specific because mutant p13 did not have the same effect. Altogether it is suggested that p13 might help keep the infected cells “normal” through selectively killing the transformed HTLV-1 cells. More studies are required before we can understand how p13 functions in the persistence of infection.

## CONCLUSION

**FIGURE 2:**
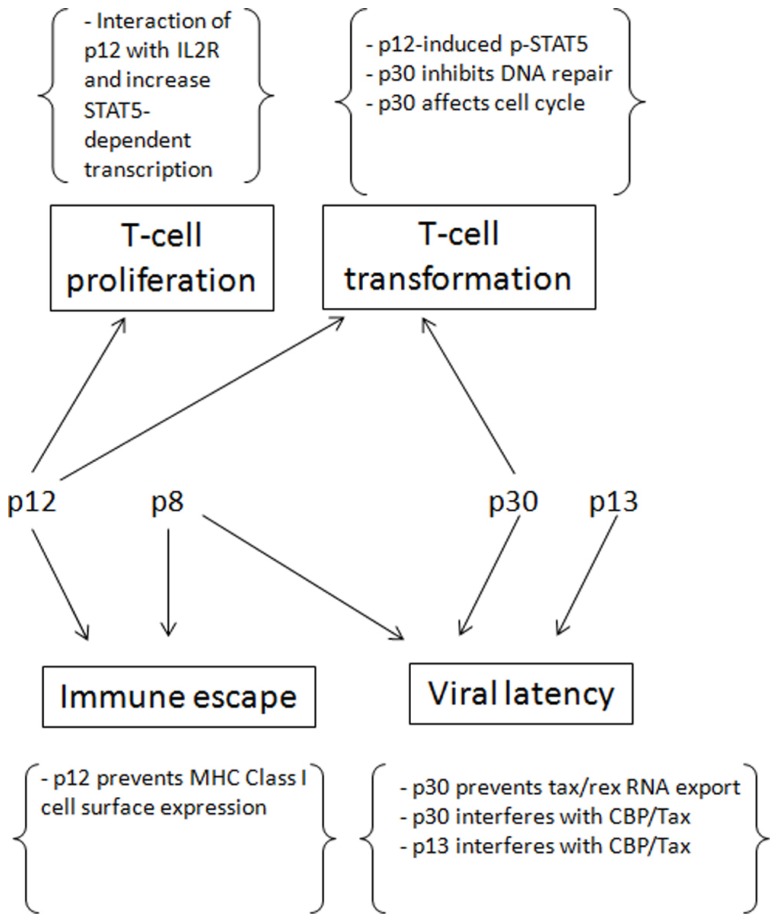
**The contributions of p12, p8, p30 and p13 for the establishment of persistent infection and cell transformation**.

Human T-lymphotropic virus type-1 persistence depends on establishment of latency. p12, p8, p30, and p13 play critical functions to help the virus to establish latency, evade immune surveillance and promote transformation (**Figure 2**). After infection and before the establishment of an effective immune response to HTLV-1, Tax and Rex can be expressed at high levels, producing as much virus as possible to infect and transform as many cells as possible. At the same time that p13, p30, and HBZ interfere with Tax-mediated transcription and reduce the total level of viral expression, p30 retains *tax/rex* RNA and reduces Tax and Rex directly. Once the host acquires immunity against HTLV-1, especially Tax, only the cells with less Tax through cell regulation and/or expression of p30, p13, and HBZ can survive. However, to maintain the infection and transform the cells, a low level of Tax expression is still required. The presence of p12 helps the infected cells to avoid being recognized by CTL or NK cells, p30 can deregulate innate immune responses, and p8 increases the efficiency of virus transmission.

In addition, p12 and p30 can compensate for the low expression of Tax by promoting T cell proliferation and increasing DNA instability, thereby transforming cells. The virus also evolves ways to counteract some negative regulations, such as HBZ decreasing p30 expression and Rex inhibiting p30 and rescuing *tax/rex* export. These measures prevent the absolute latency of the virus. In conclusion, the concerted expression of p12, p8, p30, and p13 help the virus to establish and maintain an incomplete latency in which the virus expresses low levels of viral proteins. In turn, these maintain the infection and transformation at the same time that they evade immune surveillance.

## Conflict of Interest Statement

The authors declare that the research was conducted in the absence of any commercial or financial relationships that could be construed as a potential conflict of interest.
